# The 30th anniversary of the UK Association for Computational Mechanics

**DOI:** 10.1007/s00366-023-01804-z

**Published:** 2023-03-13

**Authors:** Ruben Sevilla

**Affiliations:** Zienkiewicz Centre for Computational Engineering, Swansea University, Swansea, Wales SA1 8EN UK

**Keywords:** UK, Computational mechanics, Association, History

## Abstract

This article commemorates the third decade of the UK Association for Computational Mechanics, formerly known as the Association of Computational Mechanics in Engineering. An historical overview is provided, from the establishment of the Association in March 1992 up to the end of 2022. The article includes brief biographies of the main researchers involved in the establishment of the Association. Over this 30-year period, the Annual Conference has been a significant event and details are given of the Conference organisers, the venues and the plenary speakers.

## The catalyst

**Fig. 1:**
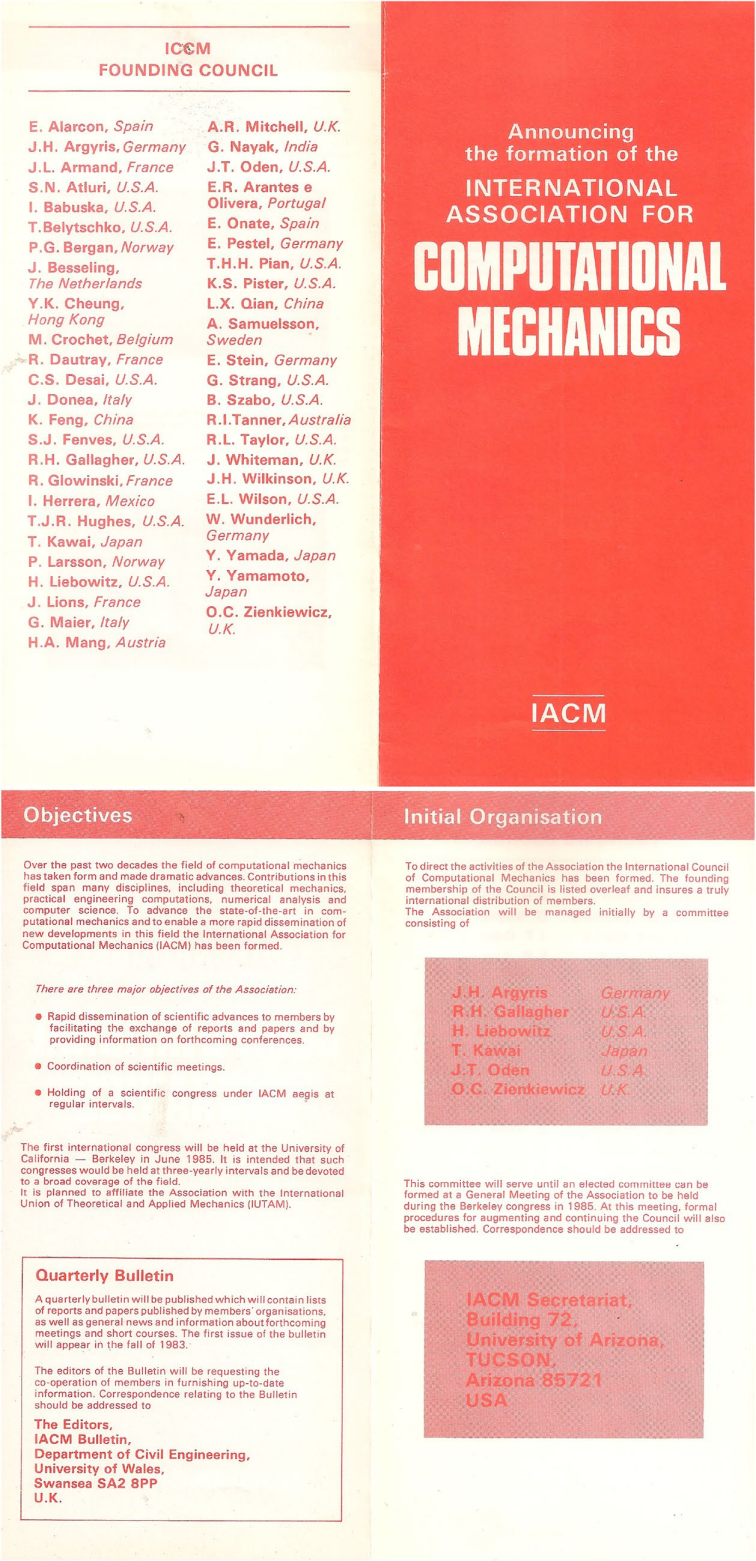
The flyer that announced the formation of IACM, supplied by Walter Wunderlich

During the 1980s, the field of computational mechanics experienced a true revolution, due to the increased capability, and availability, of computational resources. In 1981, Richard H. Gallagher, J. Tinsley Oden and Olgierd C. Zienkiewicz organised a meeting to establish the International Association of Computational Mechanics (IACM). Four UK scholars were members of the IACM Founding Council, namely Andrew R. Mitchell (University of Dundee), John Whiteman (Brunel University), James H. Wilkinson (National Physical Laboratory) and Olgierd C. Zienkiewicz (Swansea University). The flyer that announced the formation of IACM, depicted in Fig. 1 and supplied by Walter Wunderlich [1], shows the names of the scholars that formed the founding council.

IACM was initially managed by a committee formed by John H. Argyris, Richard H. Gallager, Harold Liebowitz, Tsuyoshi Kawai, J. Tinsley Oden and Olgierd C. Zienkiewicz. From 1986 to 1990, Olgierd C. Zienkiewicz served as the first president of IACM.

**Fig. 2:**
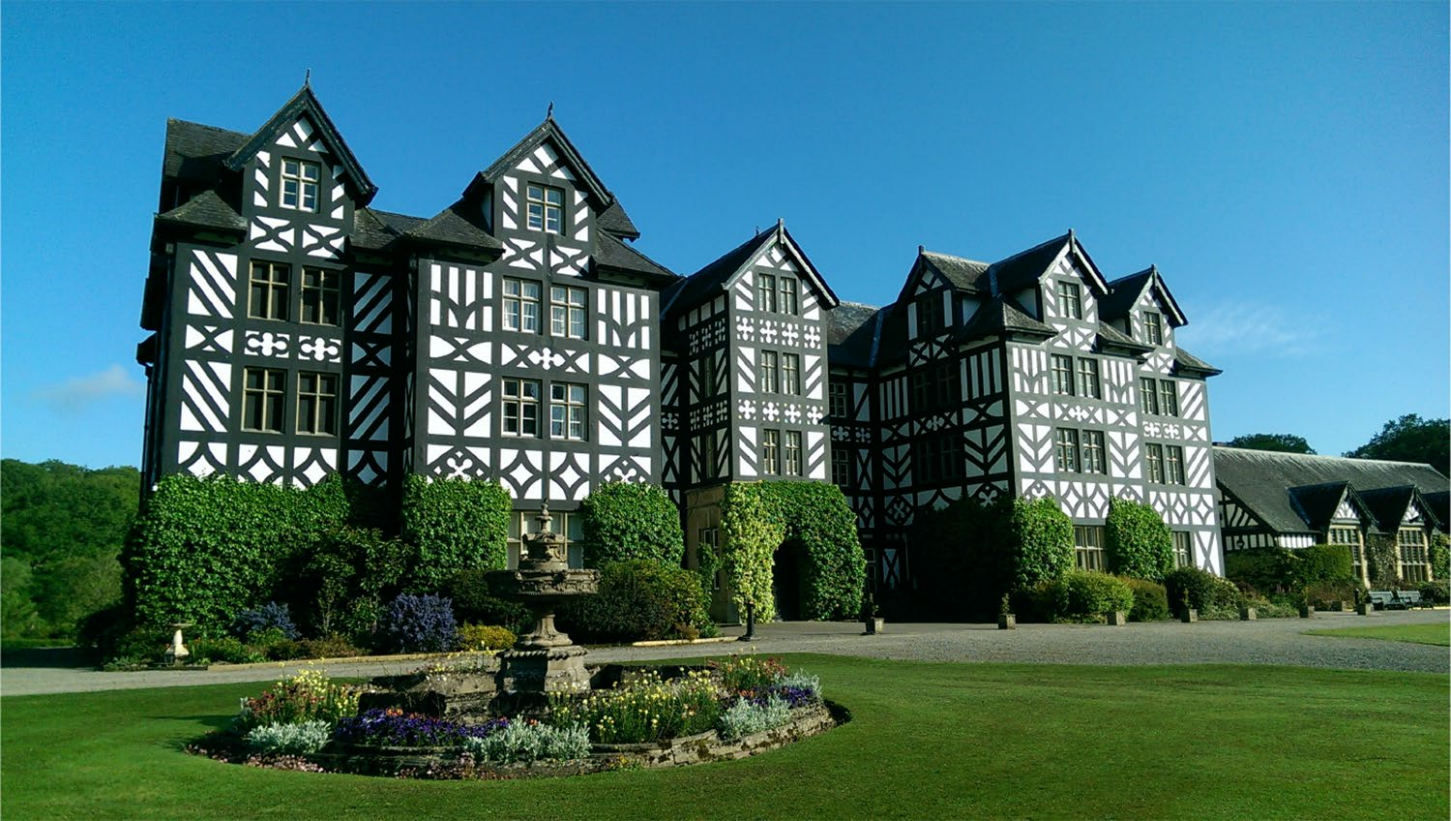
Gregynog Hall

In the late 1980s, a few meetings and technical discussions were organised at Gregynog Hall, a historic house in mid Wales, UK, shown in Fig. 2, owned by the University of Wales. These meetings proved to be the main catalyst for the formal creation of the Association in 1992.

**Fig. 3:**
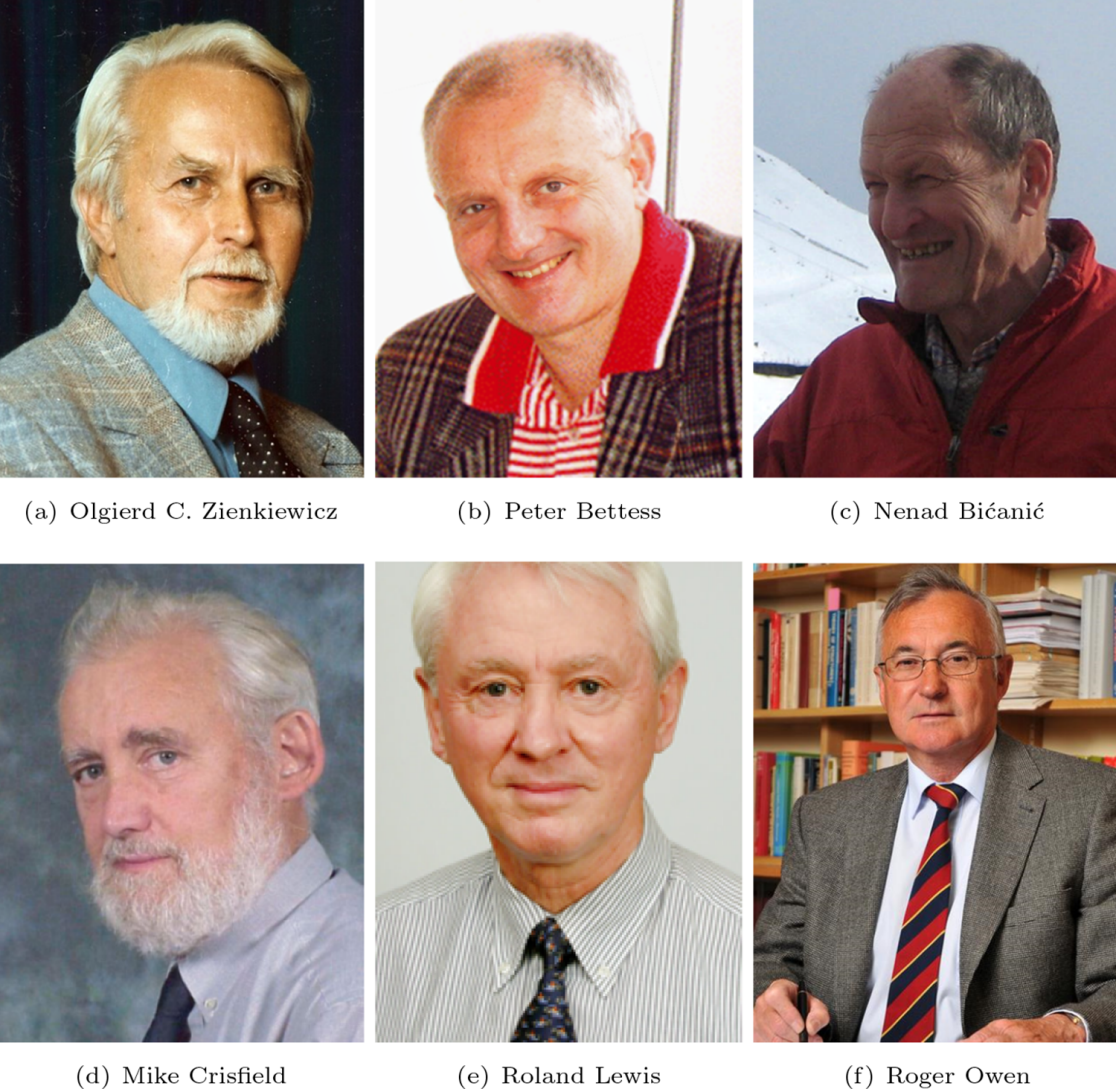
Six of the personalities that played an integral role in the creation of the Association

*Olgierd C. Zienkiewicz (1921–2009)*, portrayed in Fig. 3a, was the prime mover behind the organisation of the meetings at Gregynog. Zienkiewicz is recognised internationally as one of the leading developers of the finite element method and one of the pioneers in applying the finite element method to non-structural problems. His books on the finite element method remain a major resource for undergraduate and postgraduate courses [2, 3]. Zienkiewicz also founded, with Richard H. Gallager, the International Journal for Numerical Methods in Engineering [4, 5], the first international journal focused on the development of numerical methods. His contribution to the field of computational mechanics was immense. Not only was he involved in establishing IACM and the UK Association but also, after his retirement, he contributed to the establishment of the Spanish Association for Numerical Methods in Engineering (SEMNI) [6, 7].

Many others, who were also instrumental in the organisation of the meetings at Gregynog, were an integral part of the establishment of the Association. Among these, the five distinguished researchers that are particularly remembered for their role are Peter Bettess, Nenad Bićanić, Mike Crisfield, Roland Lewis and Roger Owen.

*Peter Bettess (1945–2020)*, portrayed in Fig. 3b, played an important role in the establishment of the Association. He completed his BSc and MSc at Imperial College and then moved to Durham to complete his Ph.D. After spending some time working for the British Ship Research Association, he became a lecturer at Swansea, in 1974, where he worked with Olgierd Zienkiewicz. In 1986, he became the Lloyd’s Register Professor of Offshore Engineering at the University of Newcastle upon Tyne. He returned to Durham in 1995, where he continued his teaching and research activities until his retirement in 2004. He made significant contributions to the field of wave propagation, including the introduction of infinite elements [8, 9].

*Nenad Bićanić (1945–2016)*, portrayed in Fig. 3c, was a leading light in the creation and continued success of the Association, moulding the Annual Conference to support young researchers. Bićanić came to the UK during a sabbatical to undertake his Ph.D. at Swansea, under the supervision of Olgierd Zienkiewicz and Ernest Hinton. In 1985, Bićanić returned to Swansea as a lecturer and was soon promoted to senior lecturer and then reader. In 1994, he was appointed to the Regius Chair of Civil Engineering at the University of Glasgow [10]. He made numerous contributions to the field of computational mechanics, in areas such as concrete mechanics, fracture, dynamics, computational plasticity, discrete element method, discontinuous deformation analysis, multi-scale mechanics and multi-physics modelling [11, 12].

*Mike Crisfield (1942–2002)*, portrayed in Fig. 3d, played a major role in the early days of the Association. Crisfield obtained his first degree and doctorate at Queen’s University in Belfast. After periods in Taylor Woodrow and the Ministry of Development in Northern Ireland, he joined the Transport and Road Research Laboratory. From there, he was appointed to a chair at Imperial College, London. Crisfield made significant contributions to the field of non-linear computational mechanics [13, 14]. He will be remembered, not only for being an eminent researcher and a true scholar, but also for being a remarkable person. His ability to light up conferences, with his particular style of presentation and his social contribution, will be always remembered by those who were lucky enough to meet him. In spite of his relatively early death, he was already recognised for the impact of his achievements [15, 16].

*Roland Lewis (1940–)*, portrayed in Fig. 3e, also played a central role in the meetings at Gregynog and in the creation of the Association. After obtaining his Ph.D. at Swansea, Lewis spent some time working in Imperial Oil Ltd Canada and Chevron Standard California, before taking up a lecturer position in Swansea in 1969. He made early contributions in the areas of soil mechanics and his book, The Finite Element Method in the Static and Dynamic Deformation and Consolidation of Porous Media, remains the state-of-the-art book in the field [17]. He is also well known for his contributions in the simulation of casting, ground freezing and heat and mass transfer. In addition to his scientific contributions, Lewis has made significant contributions as editor of renowned journals [18]. In 2019, Roland Lewis was made an Honorary Fellow of Swansea University.

*Roger Owen (1942–2020)*, portrayed in Fig. 3f, made significant contributions, not only to UKACM but also to the European Community on Computational Methods in Applied Sciences (ECCOMAS) and to the IACM. Apart from his time as a Ph.D. student and post-doctoral researcher at Northwestern University, Owen spent his whole career at Swansea University. He made leading scientific contributions in the fields of computational plasticity, working with both finite and discrete element methods [12, 19, 20]. He was the recipient of many honours, including being elected a Fellow of the Royal Society and a Foreign Member of both the US National Academy of Engineering and the Chinese Academy of Sciences. Owen was also a co-founder of Rockfield Software Ltd, a company which specialises in finite/discrete element software and received the Queen’s Award for Innovation twice. On top of his academic contributions, Owen will be remembered by his remarkably good humour and ability to socialise [21].

## Early days

**Fig. 4:**
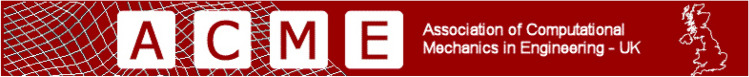
Original logo of the Association

The UK Association for Computational Mechanics was initially named the Association of Computational Mechanics in Engineering (ACME). The original logo of the Association is shown in Fig. 4.

Its mission was to both promote research in computational mechanics within the UK and to establish formal links with similar organisations in Europe and worldwide. It is worth mentioning that the European Community on Computational Methods in Applied Sciences (ECCOMAS) was founded in 1993. Professor Jacques Périaux led the establishment of ECCOMAS, recognising the need for a European Association that brought together many national associations in Europe.

**Fig. 5:**
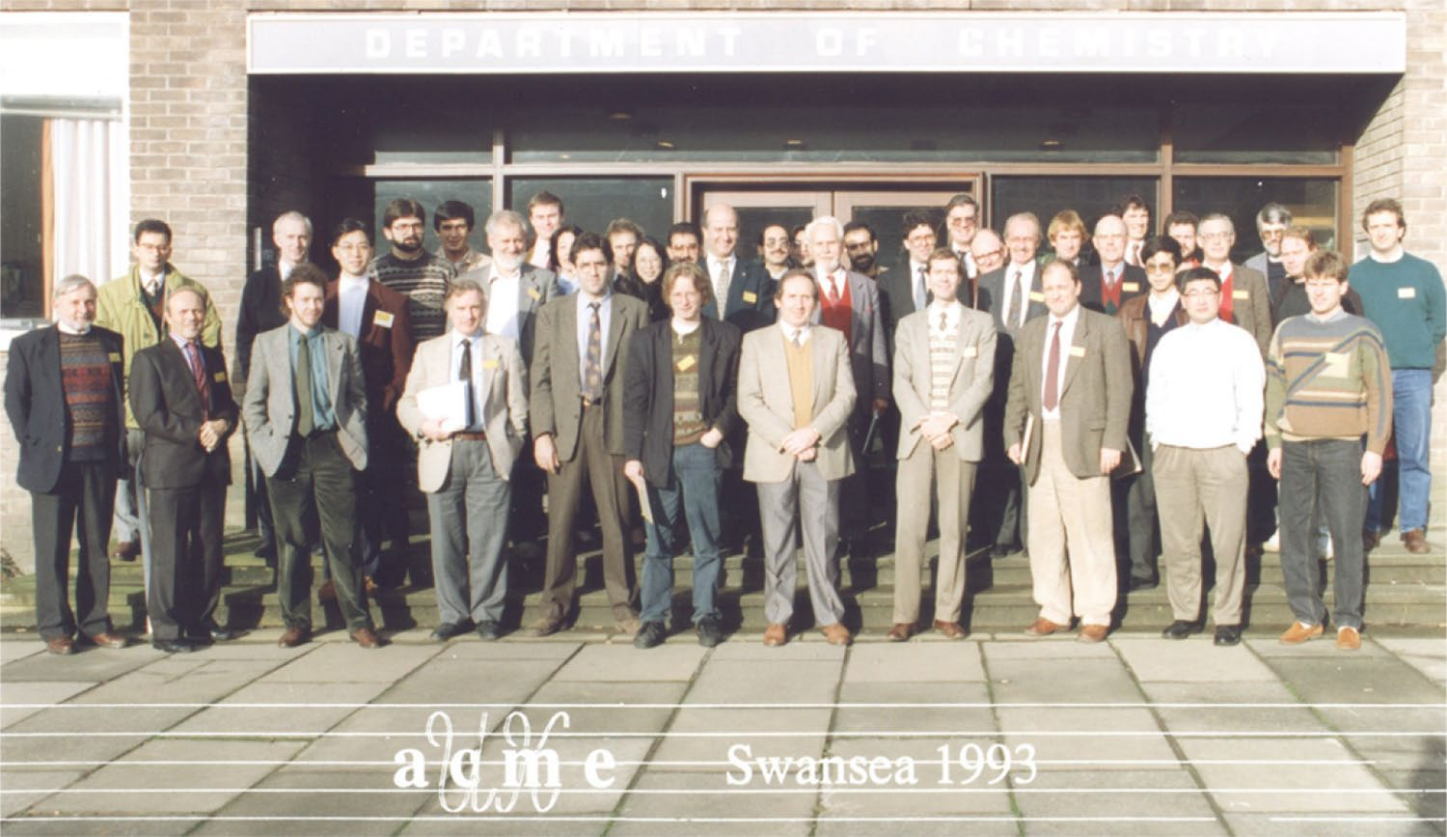
Group photo taken during the first ACME Conference held at Swansea University in 1993. Among those present here are E. de Sousa Neto, D. Nicholas, N. Weatherill, D. Peric, M. Crisfield, J. Peraire, P. Bettess, N. Verhoefen, M. Manzari, C. Taylor, O. C. Zienkiewicz, H. Afshar, A. Hutton, K. Parrott, I. Smith, B. Spalding, N. Bicanic, J. Whiteman, A.Craig

The principal activity of ACME involved the organisation of an annual two-day conference on computational mechanics, developments and research trends. Olgierd C. Zienkiewicz chaired the first ACME Conference that took place in March 1993 in Swansea and he was named honorary president for life. A group photograph taken during the first ACME Conference is shown in Fig. 5.

Following Zienkiewicz’s vision of creating a meeting targeted at early career researchers, the conferences offered the opportunity for young researchers to meet, share their ideas and build collaborative networks. At the early meetings, there were no invited speakers, only talks of 20 min in a two-day intensive conference. The proceedings appeared in the form of extended abstracts (typically four pages) and awards were given for the best student paper and for the best student presentation.

For established academics, it was an opportunity to spot talent for their departments and research groups. Many of the Ph.D. students and post-doctoral researchers who attended the early conferences are now leading professors in the field of computational mechanics.

**Fig. 6:**
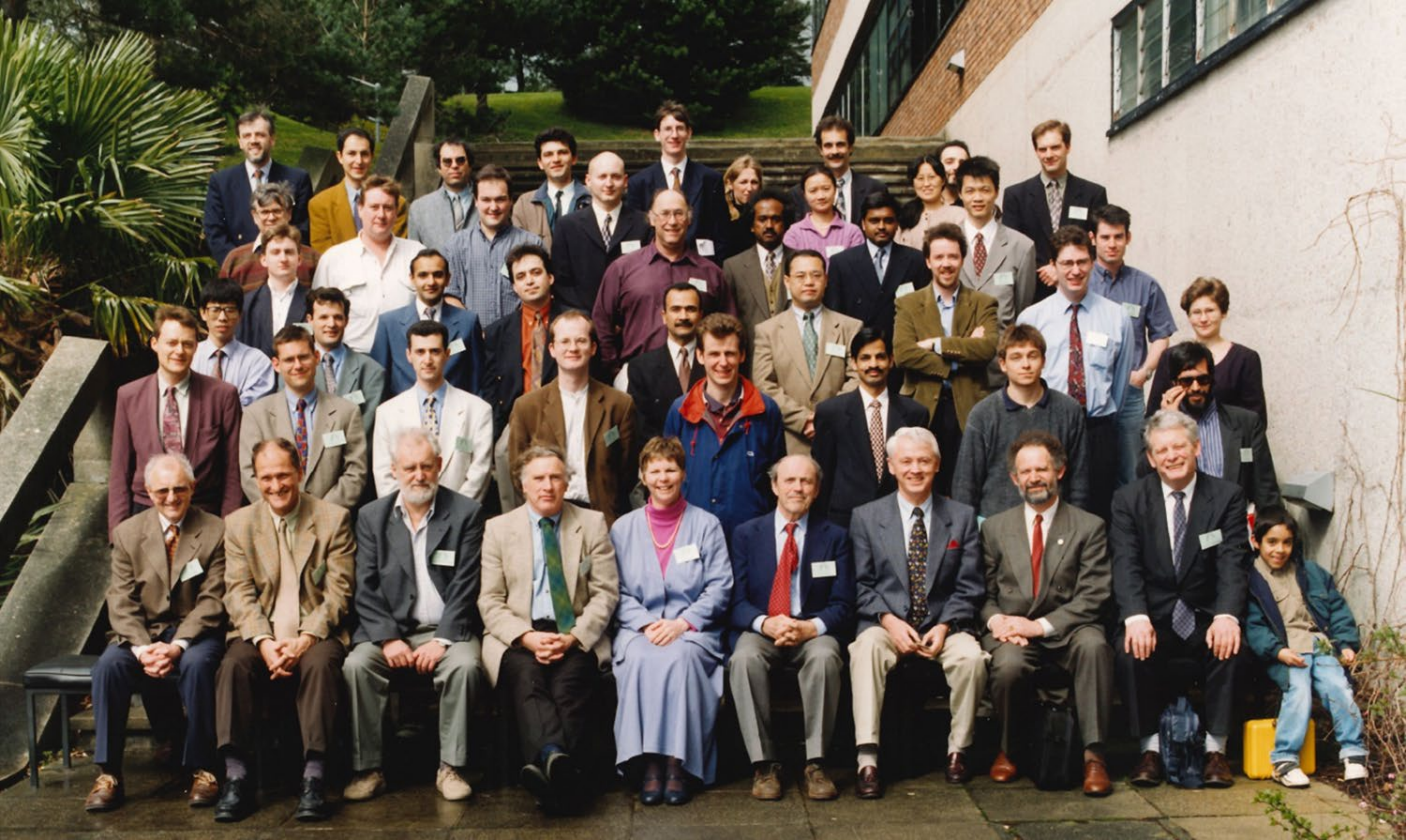
Group photo taken during the ACME Conference held at the University of Exeter in 1998. Among those present here are P. Bettess, N. Bicanic, M. Crisfield, E. Maunder, D. Nicholas, R. Lewis, O. Laghrouche, C. Pearce, J. Bonet

The next Annual Conferences were held at the University of Manchester (chaired by Ian Smith), the University of Oxford (chaired by Kevin Parrott), the University of Glasgow (chaired by Nenad Bićanić) and Imperial College London (chaired by Mike Crisfield). In 1998, 5 years after the first meeting, the Conference was organised at the University of Exeter (chaired by Edward Maunder). The group photograph taken at Exeter is shown in Fig. 6.

In 1999, the Conference was held at Durham University (chaired by Peter Bettess). The 1990s is often considered a golden age of computational mechanics. For the first time, a significant amount of the work was three dimensional and non-linear in some significant sense. The geometries were ever more complex and the enabling technologies of mesh generation and visualisation were coming to the fore. In addition, high-performance parallel clusters were being successfully exploited for the simulation of problems with meshes in excess of a million elements. Many features, that are now regarded as common place, were all coming together at that time.

**Fig. 7:**
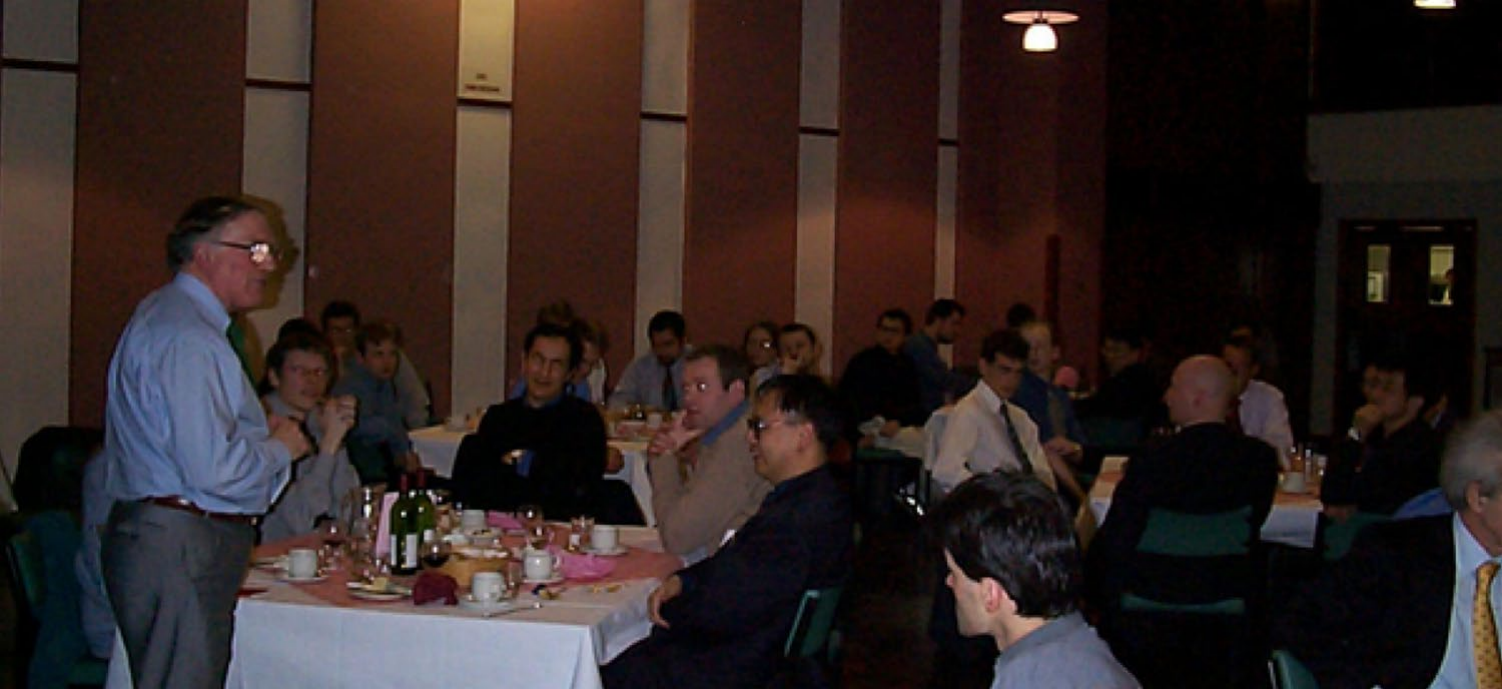
Edward Maunder delivering the after-dinner speech at the 2001 Conference at the University of Birmingham. Among those present here are A. Chan, P. Ledger

The University of Greenwich organised the Conference in 2000 (chaired by Mark Cross). Plenary talks were delivered by Olgierd C. Zienkiewicz, Swansea University, UK and Ekkehard Ramm, University of Stuttgart, Germany. The talks covered the past, present and future of computational mechanics and multi-disciplinary analysis. The 2001 Conference was organised at the University of Birmingham (chaired by Andrew Chan). An invited talk was delivered by Bernard Schreffler on the modelling of hydro-thermal and mechanical performance of a concrete tunnel during fire. The photograph of Fig. 7 shows Edward Maunder delivering the after-dinner speech.

For those who attended the early conferences, several anecdotes come readily to mind. In the 2000 Conference, organised by Mark Cross at Greenwich, Olgierd C. Zienkiewicz delivered an invited plenary talk by using a single slide, which was the cover page of the new edition of his text book. However, his talk was inspirational, with its focus on the importance of computational mechanics and the achievements of the current generation. He then spent two days giving advice to, and signing autographs for, young researchers, who were excited to be able to say that they had met the great man. Andrew Chan was known for dozing off during talks and, upon waking up during the round of applause at the end, asking the most insightful questions. The personality and sense of humour of academics, such as Mike Crisfield and Roger Owen, were also a highlight. The evenings were always relaxed affairs, where students discovered their revered professors were not quite so reverential after a drink or two.

Each year during this period, the Association was chaired by the organiser of the Annual Conference. The executive committee, of Peter Bettess, Nenad Bićanić, Andrew Chan, Mike Crisfield, Roland Lewis, Edward Maunder, Roger Owen and Ian Smith, all made significant contributions to the Association.

In 2002, the Conference celebrated the 10th Anniversary of the Association and returned to Swansea University (chaired by Javier Bonet). A special issue was created after the Conference [22] and it was dedicated to Mike Crisfield, who sadly passed in February 2002, after a short battle with cancer.

Also in 2002, both ACME and ECCOMAS started to award prizes for the best Ph.D. thesis in the UK and Europe, respectively. It is worth noting that from 2002 to 2008, three Ph.D. thesis from the UK were awarded a prize by ECCOMAS. ACME also continued to provide awards for the best presentation in the Annual Conference, for the best post-doctoral researcher and for the best postgraduate research student. This strengthened the position of the Association in providing a platform for the young generation of UK researchers in computational mechanics. The award for the best presentation was named after Mike Crisfield. This tribute was particularly well deserved, given the outstanding contributions of Mike Crisfield to the computational mechanics community, in general, and to ACME in particular. It is also a recognition of his contributions to the Conferences, where his presentations would regularly attract large audiences, eager to discover the secret of, what was called, the “Crisfield school of presentation”. A well-remembered anecdote of the time is that, following a presentation that used all the available multimedia tools, Mike Crisfield began his talk with the words “I too have a multimedia presentation. I have three coloured pens!”.

The 2003 Conference took place at the University of Strathclyde (chaired by Marcus Wheel). The Conference followed a slightly different format, with no parallel sessions or plenary lectures. The presentations covered a variety of topics, such as the analysis of soils and porous media, forming processes, computational fluid dynamics, surface representation, meshing and conceptual design.

In 2004, the Annual Conference was organised at Cardiff University (chaired by Hywel Thomas). The 13th Annual Conference, hosted by the University of Sheffield (chaired by Roger Crouch), incorporated a social walk the day before the Conference and a special session delivered by the Manchester High Performance Computing Unit and EPSRC was included in the programme. Four plenary lectures were delivered by renowned UK researchers. Marian Wiercigroch, from Aberdeen University, delivered the first, on computing dynamic responses of non-smooth dynamical systems. Nick Higham, from the University of Manchester, talked about solving polynomial eigenproblems by linearization. Ian Smith from the University of Manchester, talked about parallel three-dimensional finite element analysis of excavation. Finally, Harm Askes, from Sheffield University, delivered a plenary on configurational forces in optimisation algorithms.

In addition to the Annual Conference, a number of other activities, organised by members of the Association, were proliferating in the UK. For instance, in 2003, a summer school on high performance computing in finite element analysis was organised at the University of Manchester (chaired by Ian Smith) and a workshop on current challenges in the integration of computational modelling and micro/nano characterisation of cementitious materials was organised at the University of Glasgow (chaired by Nenad Bićanić). In 2005, the IACM Conference on finite elements for flow problems was hosted by Swansea University (chaired by Oubay Hassan). The Association was also active in coordinating the nominations of EPSRC peer review college members within the computational mechanics community.

The 2006 Conference took place at Queen’s University Belfast (chaired by Cecil Armstrong) and was a joint effort between ACME and the Irish Society for Scientific and Engineering Computation (ISSEC). The first plenary lecture was delivered by Mike Lawson, from Rolls-Royce plc, and explored the limits of simulations. The second plenary lecture, by Antonio Rodrìguez-Ferran from Universitat Politècnica de Catalunya, Spain, focused on nonlocal inelasticity based on nonlocal displacements.

In 2007, the Conference was organised by the University of Glasgow (chaired by Barry Topping). In addition to the established prizes, a new prize, provided by Computational Technology Solutions of Stirling, was presented for the best written paper with a first named author under the age of 35. The first plenary lecture was delivered by Mark Cross, from Swansea University, UK, on the use of mixed discretisation schemes in multi-physics simulation. Nenad Bićanić, from Glasgow University, UK, delivered the second plenary lecture on similarities and differences in discontinuous modelling. Kurt Martin, from the Federal Armed Forces University Munich, Germany, gave a plenary lecture on stochastic structural optimisation with quadratic loss functions. Joño António Teixeira de Freitas, from Universidade de Lisboa, Portugal, presented a plenary lecture on the topic of mixed and hybrid stress elements for biphasic media. The fifth plenary lecture was delivered by Harm Askes, from Sheffield University, UK, who presented a formulation and finite element implementation of dynamically consistent gradient elasticity. Papers were published, after the Conference, in a special issue of the journal *Computers & Structures* [23].

In 2007, the activity within the association increased significantly, as shown by the monthly communications circulated via the ACME mailing list. ACME members were actively involved in organising other computational mechanics activities. For instance, the EPSRC Summer School on Mathematical Modelling & Computational Methods in Solid Mechanics was hosted by Glasgow University (organised by Chris Pearce), the 2007 Parallel Programming Summer School was hosted by Manchester University (organised by Lee Margetts) and the EPSRC Summer School on Continuum Solid Mechanics took place in Nottingham University (organised by Carlo Sansour).

The 2008 Conference was held at Newcastle University (chaired by Mohamed Rouainia). Plenary lectures were delivered by Kenneth Morgan, from Swansea University, UK, and Manuel Pastor, from Cedex, Spain.

## New structure and management

During a meeting of the Executive Committee, held as part of the 2009 Conference at the University of Birmingham (chaired by Carlo Sansour), Nenad Bićanić, Harm Askes and Chris Pearce submitted a proposal to modify the organisation of the Association. This was finally approved in May 2009 and resulted in several changes to the structure and management. These changes incidentally coincided with the death of Olgierd Zienkiewicz, in January 2009.

A new Executive Committee was created, consisting of the president, the secretary, the treasurer, the chair of the previous Conference and the chair of the current Conference. Carlo Sansour (University of Nottingham) was appointed as the first president, Andrew Chan (University of Birmingham) was appointed secretary and Barry Topping (Civil-Comp Ltd) was appointed treasurer.

The Board, which was also created as part of the new structure, consisted of individuals representing active UK institutions in the field of computational mechanics, members of the Executive Committee and representatives of IACM and ECCOMAS. The members of the first Board were Ferri Aliabadi, Cecil Armstrong, Harm Askes, Chris Bailey, Nenad Bićanić, Javier Bonet, Andrew Chan, Roger Crouch, Michael Hicks, Alojz Ivankovic, Tony Jefferson, Roland Lewis, Edward Maunder, Roger Owen, Chris Pearce, Mohammed Rouainia, Carlo Sansour, Barry Topping, Marcus Wheel and Antonis Zervos. In 2010, Charles Augarde replaced Roger Crouch and Akbar Javadi replaced Edward Maunder. In addition, Ante Munjiza, Omar Laghrouche and Zhenjun Yang joined the Board. Some of these names will still be familiar, even to the youngest Ph.D. student attending the latest Annual Conference, as they are still contributing to the development and day-to-day activities of the Association.

Although, at the time of the re-structuring, there were discussions within the Board about the nature of the Annual Conference, recent attendees will have noticed that the focus still follows the initial Zienkiewicz vision of providing a conference targeted at young researchers.

The 2010 Conference was held at the University of Southampton (chaired by Antonis Zervos). Plenary talks were delivered by Michel Jean, from the Laboratoire de Mecanique et d’Acoustique, Marseille, France, Nick Koutsabeloulis, from the Schlumberger Reservoir Geomechanics Centre of Excellence, UK and Paul Tucker, from Cambridge University, UK. The topics covered in the plenary lectures were non-smooth contact dynamics, computational geomechanics and computational aeroacoustics.

In 2011, the Conference was organised in Heriot-Watt University (chaired by Omar Laghrouche). Prior to the Conference, and following a proposal by Nenad Bićanić and other ACME members, an ACME School was organised for the first time. This is now a continuing tradition. Harm Askes (University of Sheffield), Charles Augarde (Durham University) and Jon Trevelyan (Durham University) lectured at the first School and covered topics such as meshless methods, the scaled boundary method and the boundary element method. The opening lecture was delivered by Roger Owen on the challenges in the modelling of particulates and multi-fracturing materials with coupled field effects.

**Fig. 8:**
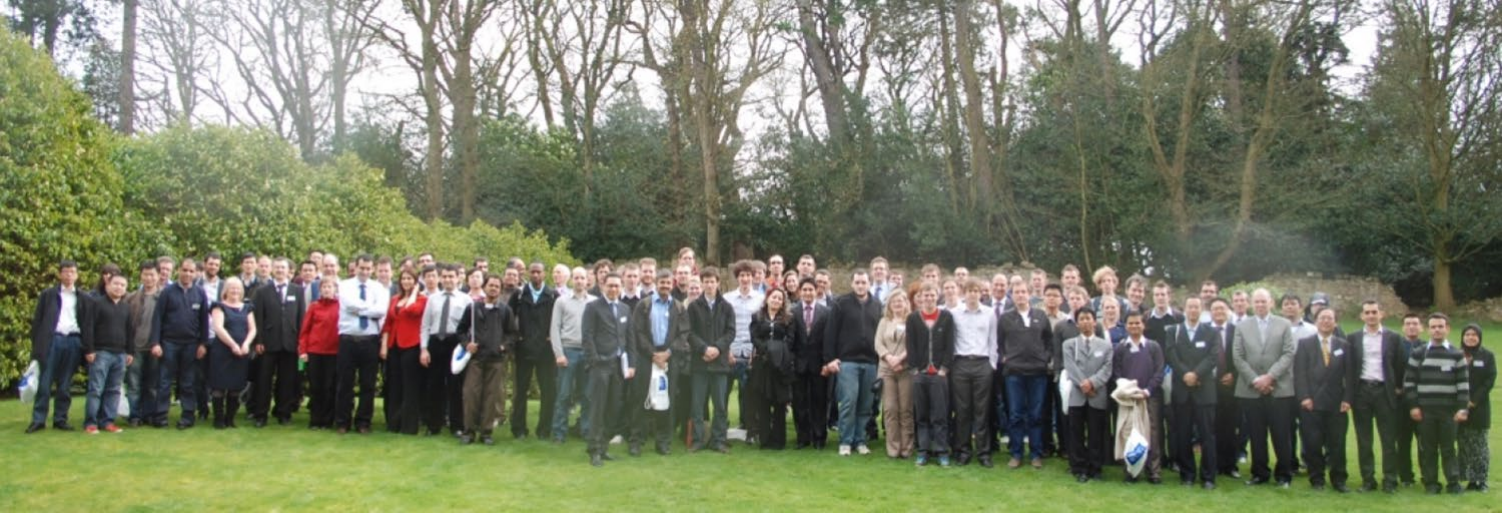
Group photo taken during the ACME Conference that took place at Heriot-Watt University in 2011. Among those present here are O. Laghrouche, M. Mehravar, A. Ahangar-Asr, R. Simpson, C. Augarde, W. Coombs, B. Topping, A. Chan, C. H. Lee, Z. Ullah, T. Jefferson, J. Trevelyan, A. Zervos, C. Sansour

The group photograph taken at Heriot-Watt University, shown in Fig. 8, shows the increased number of ACME participants. Following the Conference, papers were published in a special issue of the journal *Computers & Structures* [24].

In 2012, the Conference took place at Manchester University (chaired by Zhenjun Yang). Invited plenary lectures were given by Guirong Liu from Cincinnati, USA on smoothed finite element methods, Chongmin Song from Sydney, Australia on scaled boundary finite element method, Kenneth Morgan from Swansea, UK on adaptive remeshing of CFD problems, and René de Borst from Glasgow, UK on multiscale fracture modelling. The Conference was preceded by the 2nd ACME School, at which lectures were given by Carlo Sansour from Nottingham University, Stephane Bordas from Cardiff University and Dongfang Liang from Cambridge University. The lectures covered topics on extended finite elements, generalised continua and scaled effects, computational fluid mechanics and wave impact on structures.

**Fig. 9:**
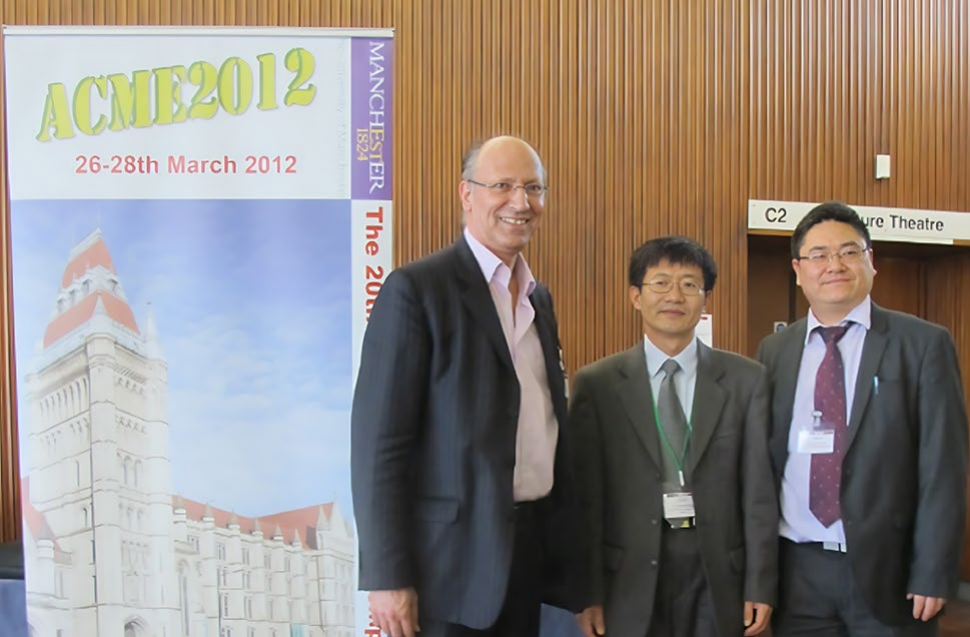
Carlo Sansour, Chongmin Song and Zhenjun Yang at the 2012 Conference at Manchester University

Figure 9 is a photograph taken during the 2012 Conference, featuring the ACME president, Carlo Sansour, the Conference organiser, Zhenjun Yang, and one of the plenary speakers, Chongmin Song. After the Conference, papers were published in a special issue of the journal *Computers & Structures* [25].

**Fig. 10:**
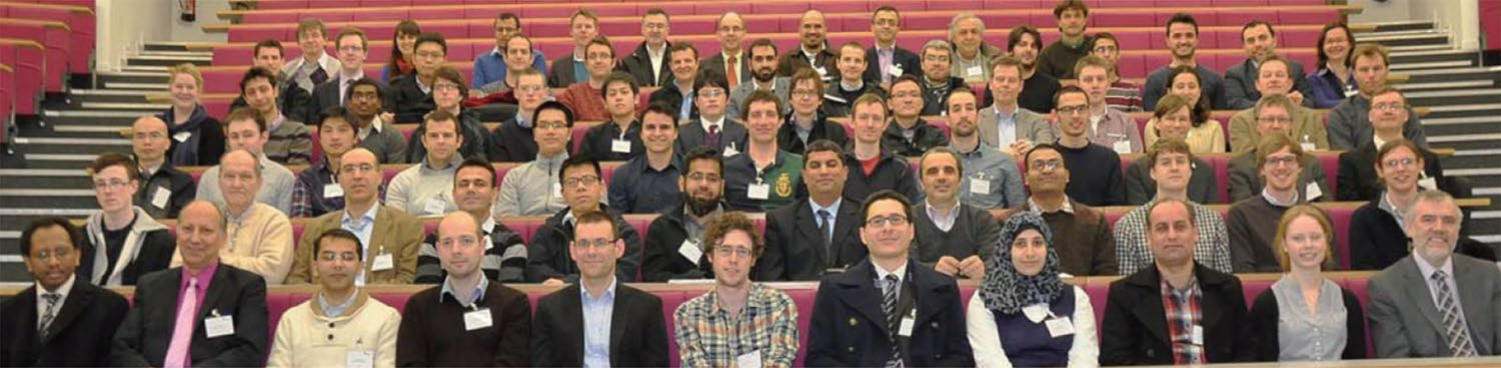
Group photo taken during the 2013 Conference that took place at Durham University. Among those present here are A. Osman, C. Sansour, C. Pearce, C. Armstrong, T. Jefferson, L. Kaczmarczyk, R. de Borst, C. Augarde, J. Bonet, W. Coombs, O. Laghrouche, J. Trevelyan, A. Javadi

In 2013, the Conference was organised at Durham University (chaired by Ashraf Osman). This Conference followed a slightly different format, with no School and with ample time allocated within the programme for formal and informal discussions. It was called the International Conference on Computational Mechanics (CM13) and it was first international conference organised under the auspices of ACME. Plenary lectures, delivered by René de Borst and Javier Bonet, covered different aspects of non-linear computational mechanics. The group photograph taken at Durham University during the Conference is shown in Fig. 10.

**Fig. 11:**
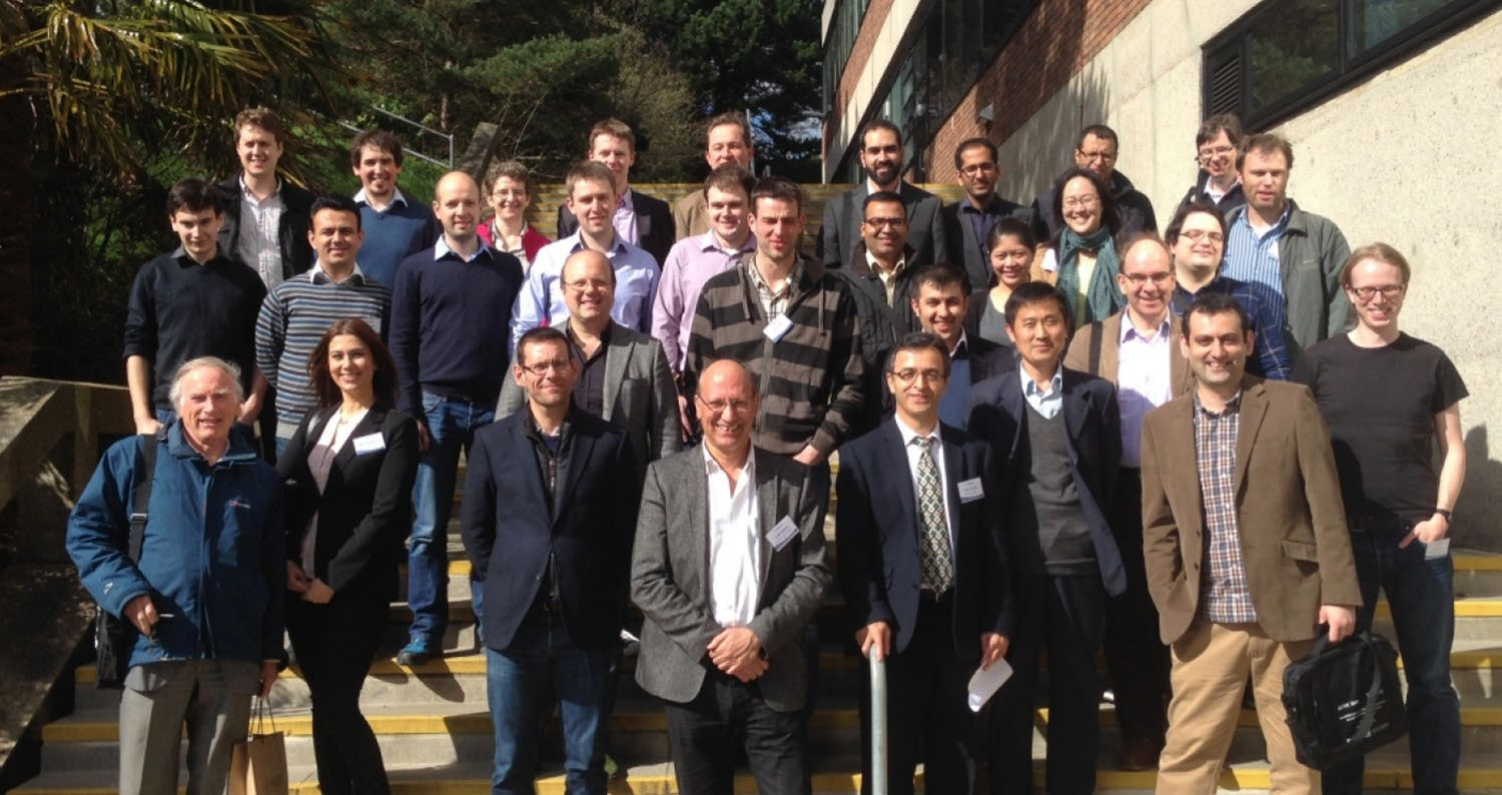
Group photo taken during the 2014 Conference that took place at Exeter University. Among those present here are E. Maunder, M. Mehravar, C. Pearce, C. Sansour, A. Javadi, A. Faramarzi, Z. Ullah, J. Trevelyan, A. Kim, W. Coombs, C. Augarde, L. Kaczmarczyk

In 2014, the Conference was held at Exeter University (chaired by Akbar Javadi). The School was delivered by Sven Klinkel, Ido Akkerman, Robert Simpson and Philippe Young, covering topics of geometry, mesh generation and finite element analysis for solids and fluids. Plenary lectures were delivered by Hywel Thomas, Adrian Gaylard and Bassam Izzuddin and addressed topics in geoenergy, automotive aerodynamics and high-performance computing for non-linear structural analysis. The group photograph taken at Exeter University during the Conference is shown in Fig. 11.

The final Conference of this period, held in Swansea University (co-chaired by Antonio Gil and Rubén Sevilla), involved, as plenary speakers, illustrious researchers who had started their career in the UK, namely Luca Formaggia, Rainald Löhner and Jaume Peraire, as well as Jörg Schröder, from Universität Duisburg-Essen, Germany. A prize sponsored by SIAM and a prize sponsored by the NRN were awarded, in addition to the traditional Conference prizes. A research highlight competition was organised, prior to the Conference, and the winner was offered free registration to the 2015 Conference. The ACME School was delivered by Kenneth Morgan, Paul Ledger and the Conference organisers, covering topics on low and high-order methods for solids, fluids and electromagnetics.

**Fig. 12:**
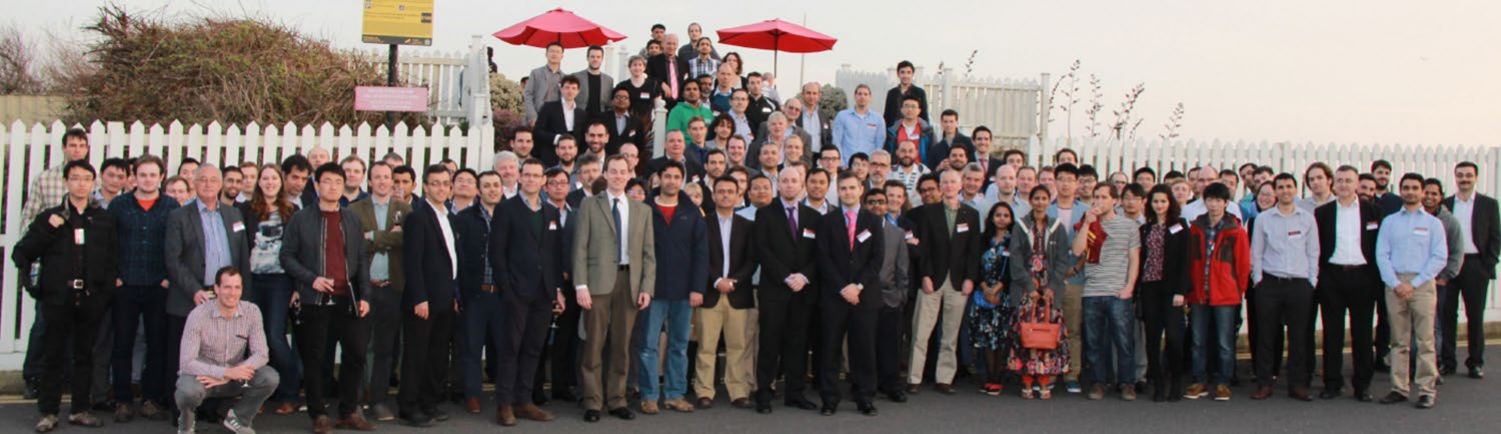
Group photo taken during the 2015 Conference that took place at Swansea University. Among those present here are R. Owen, S. Claus, A. Javadi, C. Pearce, Z. Ullah, R. Sevilla, A. Gil, C. Kadapa, R. Löhner, O. Laghrouche, J. Schröder, D. Perić, C. H. Lee, J. Bonet, S. Adhikari, J. Peraire, C. Augarde, W. Coombs, K. Morgan, J. Peiró, L. Formaggia, O. Hassan, C. F. Li, P. Nithiarasu, M. Edwards, C. Sansour

The group photograph taken at Swansea University is shown in Fig. 12. Following the Conference, papers were published in special issues of the journal *Computers & Structures* [26], the *European Journal of Computational Mechanics* [27] and the *Proceedings of the Institution of Civil Engineers-Engineering and Computational Mechanics* [28].

## Consolidation

In 2016, Charles Augarde (Durham University) took over the role of president with Akbar Jabadi (the University of Exeter) as secretary and Omar Laghrouche (Heriot-Watt University) as treasurer. During his term in office, Charles Augarde provided the foundation for a more professional functioning of the Association. The name of the Association was changed to the UK Association for Computational Mechanics (UKACM), the UKACM Bylaws were established and new policies were established for UKACM membership, UKACM prizes and UKACM support. Soon after his appointment, Charles Augarde recognised the importance of a strong online presence of UKACM. Rubén Sevilla (Swansea University) was appointed webmaster and invited to join the Executive Committee.

**Fig. 13:**
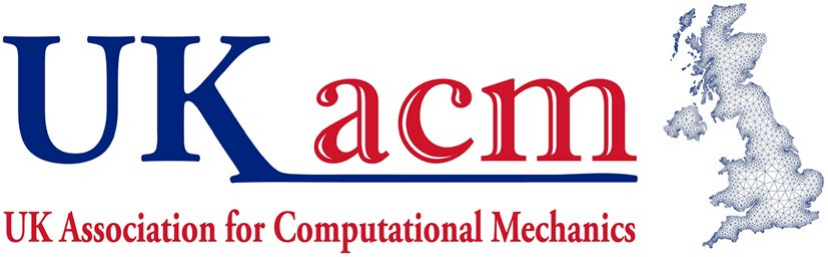
UKACM logo designed after the rebrand of the Association

A new website was created, a new logo was designed, shown in Fig. 13, and UKACM entered the modern world of social media. The visibility of the Association was maximised, both in the UK and worldwide, and the activities of the young generation of UK researchers in computational mechanics were promoted, by allowing them to publish research highlights. Vacant positions in the UK, and papers published by UK researchers, were also highlighted in this way.

By the end of his term in office, Charles Augarde had also created the Executive Committee posts of prize coordinator (Antonis Zervos, the University of Southampton) and ECCOMAS YIC representative (Will Coombs, Durham University). Four more institutions had also joined the UKACM Board. In addition, René de Borst (University of Sheffield), Chris Pearce (University of Glasgow), Harm Askes (University of Sheffield) and Roger Owen (Swansea University) were co-opted as Executive Committee members, in recognition of their involvement in IACM and ECCOMAS.

In 2016, the Conference took place at Cardiff University (chaired by Tony Jefferson and Pierre Kerfriden). This was the last ACME Conference, with subsequent meetings carrying the UKACM branding. This Conference was preceded by a School, delivered by the Conference organisers, that covered topics in nanomechanics, micromechanics, computational upscaling and homogenisation and model order reduction techniques. The plenary talks were delivered by Antonio Huerta, from Universitat Politècnica de Catalunya, Spain, Anthony Gravouil, from Institut National des Sciences Appliquées de Lyon, France, Bert Sluys, from TU Delft, The Netherlands and Garth Wells, from the University of Cambridge, UK. The end of 2016 was sadly marked by the death of Nenad Bićanić, who passed away in October 2016.

**Fig. 14:**
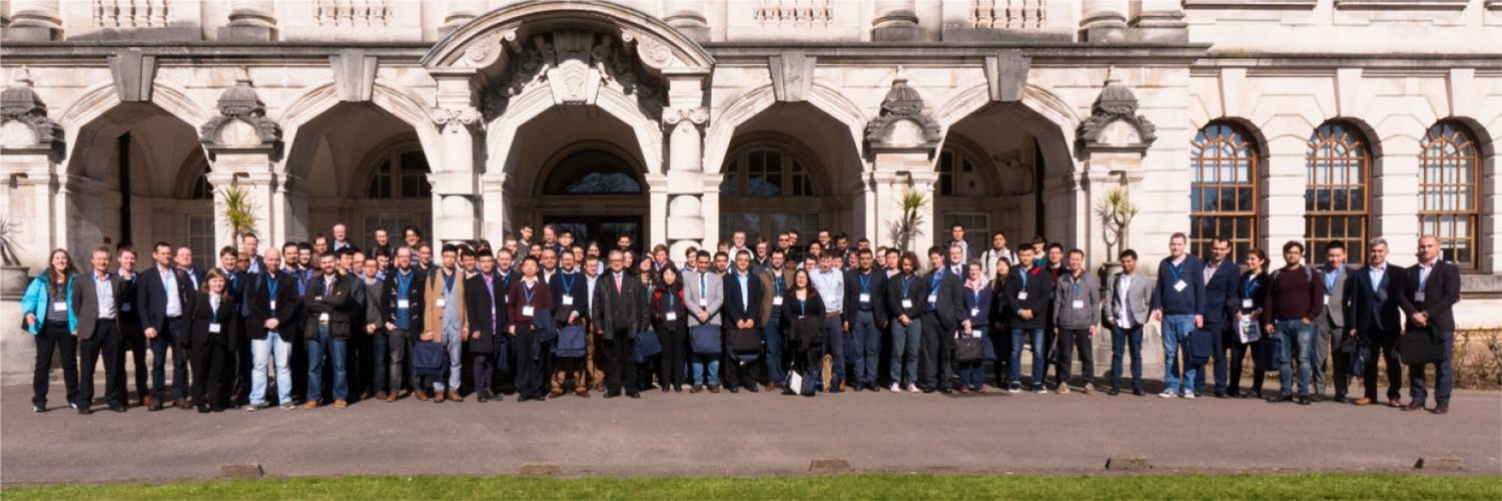
Group photo taken during the 2016 Conference that took place at Cardiff University. Among those present here are S. Claus, T. Jeffersion, W. Coombs, C. Pearce, C. Augarde, R. Sevilla, A. Gil, G. Wells, A. Huerta, A. Javadi, P. Kerfriden, M. Mehravar, A. Faramarzi, O. Laghrouche

The group photograph taken at Cardiff University is shown in Fig. 14.

**Fig. 15:**
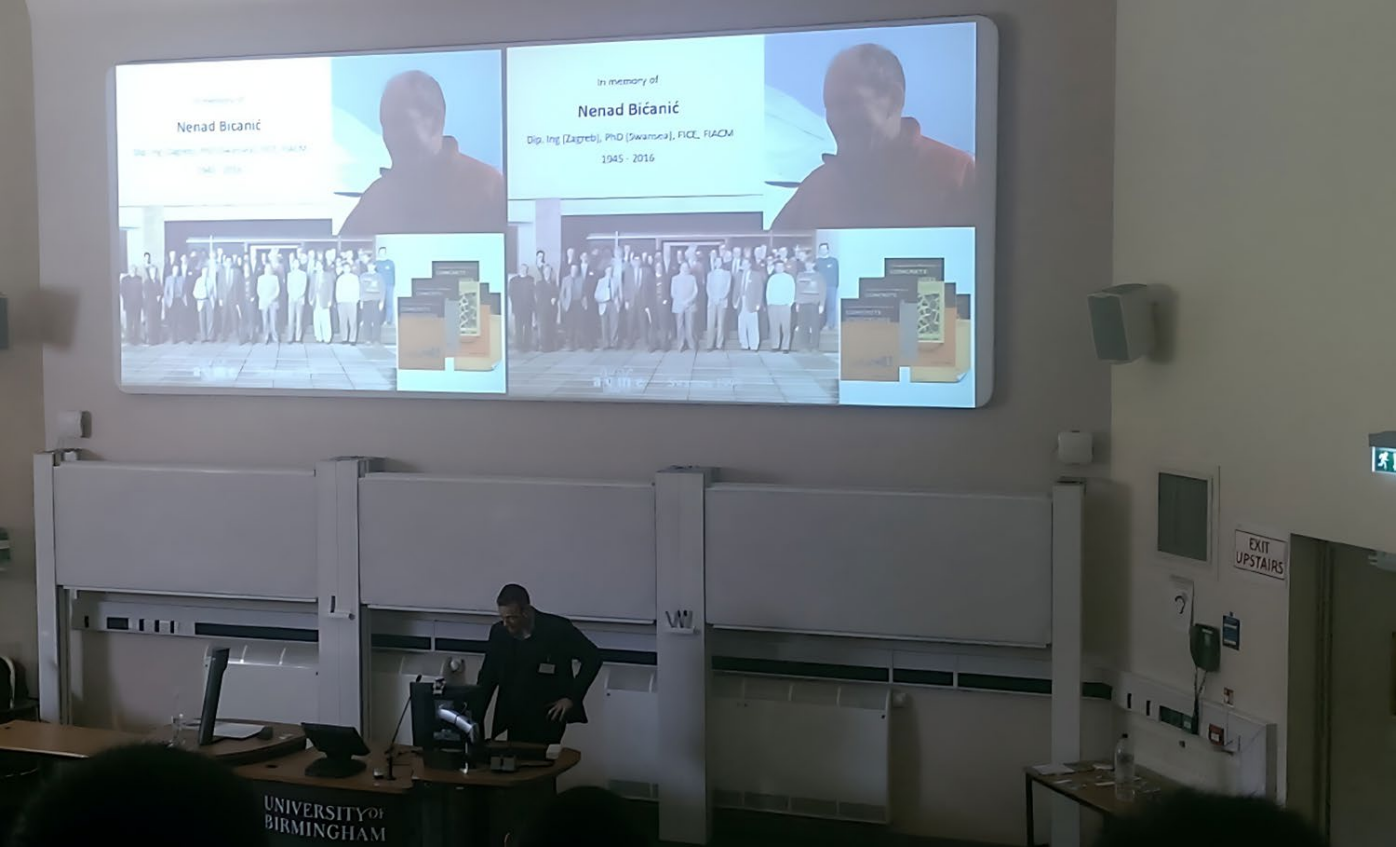
Chris Pearce during the opening of the 2017 Conference

In 2017, the Conference took place at the University of Birmingham (chaired by Asaad Faramarzi). The UKACM School incorporated a session, sponsored by COMSOL, in which participants had the opportunity to get hands-on experience with software, before attending lectures on train aerodynamics and large-scale topology optimisation, delivered by Chris Baker, Hassan Hemida and Michal Kocvara. During the opening of the Conference, Chris Pearce (University of Glasgow) dedicated the Conference to the memory of Nenad Bićanić, as depicted in Fig. 15. Fehmi Cirak, Spencer Sherwin and Mike Hartnett delivered plenary talks at the Conference.

**Fig. 16:**
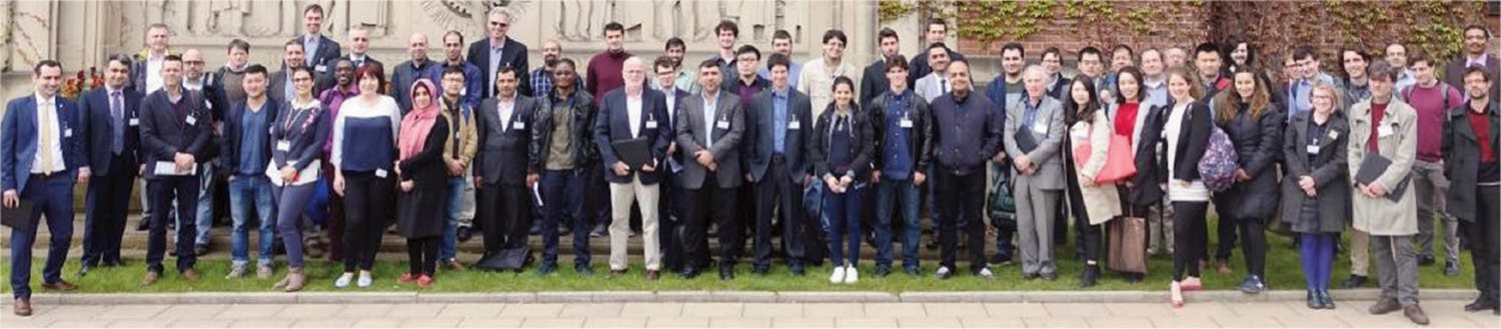
Group photo taken during the 2017 Conference held at the University of Birmingham. Among those present here are A. Faramarzi, A. Ahangar-Asr, C. Pearce, M. Mehravar, C. Baker, H. Hemida, P. Kerfriden, O. Laghrouche, F. Cirak, R. Sevilla, S. Sherwin, L. Kaczmarczyk, C. Augarde

The group photograph taken at the University of Birmingham is shown in Fig. 16.

To celebrate the 25th Anniversary of ECCOMAS, the 6th European Conference on Computational Mechanics and the 7th European Conference on Computational Fluid Dynamics were jointly held in Glasgow and absorbed the 2018 UKACM Conference. The meeting was organised by the University of Glasgow and the University of Edinburgh, in partnership with UKACM, and co-chaired by René de Borst (University of Sheffield) and Jason Reese (University of Edinburgh). Roger Owen (Swansea University) was the Honorary Chairman. The Conference was a great success, with over 1,900 delegates from 52 countries.

In 2019, Rubén Sevilla (Swansea University) took over the role of President, with Tony Jefferson (Cardiff University) as secretary, Omar Laghrouche (Heriot-Watt University) as treasurer, Antonis Zervos (University of Southampton) as prize coordinator, Marco Discacciati (Loughborough University) as webmaster and Will Coombs (Durham University) as ECCOMAS YIC representative.

During this period, UKACM continued to consolidate its position, both in the UK and internationally. Following an election process, involving all the UKACM Board, four UK academics were elected as members of the ECCOMAS technical committees.

March 2019 was a very sad month for UKACM, with the loss of Jason Reese. His sudden death, less than a year after he co-chaired the ECCOMAS 2018 Conference in Glasgow, was a bitter blow. He was an authority on modelling, analysis and simulation of multiscale fluid systems and had been a very active member of ECCOMAS.

**Fig. 17:**
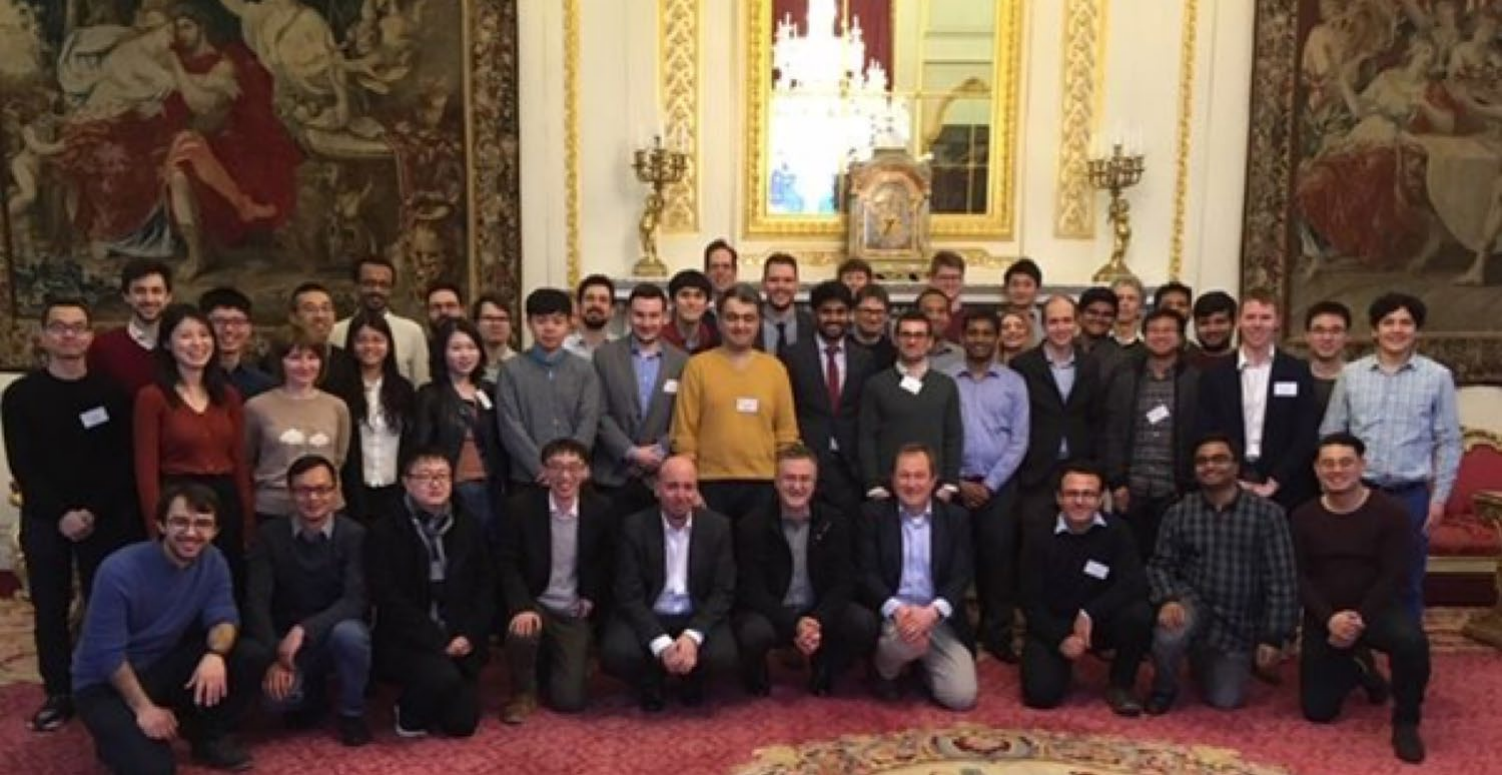
Group photo taken during the 2019 Conference held at City, University of London. Among those present here are R. Sevilla, O. Laghrouche, C. Augarde, Z. Ullah, C. Kadapa, A. Ahangar-Asr, E. Martínez-Pañeda, W. Coombs, L. Kaczmarczyk, R. Crouch

The 2019 Conference was organised at City, University of London (chaired by Roger Crouch). This Conference was characterised by a novel approach, with no parallel sessions or plenary lectures, in which participants were encouraged to deliver their presentations using the Crisfield style, with three coloured pens. A new award was made to the best presentation using this style. It is worth noting that Roger Crouch has been, to date, the only academic to have chaired two Conferences. The group photograph taken during the 2019 Conference is shown in Fig. 17.

The year 2020 brought, unfortunately, more sad news to UKACM. In January 2020, Roger Owen passed away after a long illness. Roger Owen was an integral part of the Association from the outset and he was extremely active in ECCOMAS and IACM. Soon after his death, the executive committee of UKACM unanimously agreed that the prize for the best Ph.D. thesis should now be known as The Roger Owen Prize. In March 2020 Peter Bettes also passed away. As mentioned earlier, Peter Bettess played an important role in the early days of the Association.

As readers can surely remember, 2020 also brought the disruption of COVID-19. The 2020 Conference, planned to be held in Loughborough University, was cancelled due to the pandemic. As a result, Rubén Sevilla launched an initiative to engage the young generation of UK researchers and provide a virtual platform for the dissemination of their research. A virtual research highlight competition was organised, in which participants submitted a short video presenting their research using a single slide. Virtual proceedings were also published from the abstracts submitted to the Conference.

The 2021 Conference was organised using a virtual format by Loughborough University (chaired by Marco Discacciati). Despite it being the first ever UKACM Conference organised using such a format, the School and Conference were a success, with more than 80 participants. The UKACM School was delivered by the team of the President of the Spanish Society of Numerical Methods in Engineering and the MOFEM team from Glasgow University. During the opening of the Conference, Rubén Sevilla dedicated the 2021 Conference to the memory of Roger Owen. The plenary talks were delivered by Joaquim Peirò, Imperial College London, UK, Elías Cueto, from Universidad de Zaragoza, Spain, Paola F. Antonietti, from Politecnico di Milano, Italy.

During the executive committee meeting held on April 2021, Rubén Sevilla was re-elected as UKACM president for another term.

## The 30th anniversary

**Fig. 18:**
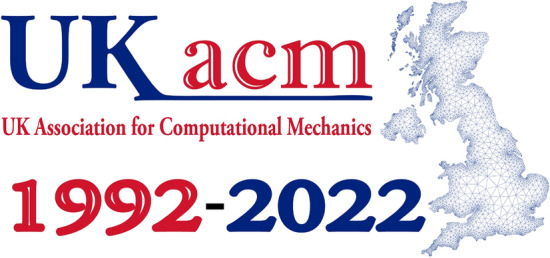
The UKACM logo designed to commemorate the 30th anniversary

Rubén Sevilla’s second term of office started in January 2022 and coincided with the 30th anniversary of UKACM. A commemorative logo, shown in Fig. 18, was designed in celebration of the anniversary.

This period has been characterised by the growth of the UKACM Board which, by the end of 2022, is now formed from 35 UK institutions, with a focus on equality and diversity and on attracting new talent to important positions within the Association.

During this period, UKACM has seen, for the first time a female chairing the National Conference and a female joining the Executive Committee. By the end of 2022, 30% of the Executive Committee members were female and the Chair of the forthcoming 2023 Conference is also female. Furthermore, Rubén Sevilla launched an initiative to name two of the Conference prizes after renowned female engineers. After consultation with the Executive Committee, the Board and the Women’s Engineering Society, in 2022 it was agreed that the UKACM prize for the best presentation by a post-doctoral researcher will be named after Nina Cameron Graham, while the UKACM prize for the best presentation by a postgraduate researcher will be named after Laura Annie Wilson.

New appointments to the UKACM Executive Committee were Chun Hean Lee (Glasgow University) as secretary, Irene Moulitsas (Cranfield University) as webmaster, Emilio Martínez-Pañeda (Imperial College London) as ECCOMAS YIC representative and Jose Luis Curiel-Sosa (Sheffield University) as prize coordinator.

The 2022 Conference was organised at Nottingham University (chaired by Jelena Ninic, Kris van der Zee, Matteo Icardi and Fangying Wang). The School, delivered by Kris van der Zee, Dominik Schillinger and Ender Özcan, covered topics such as computational phase-modelling, cut finite element methods and optimisation using metaheuristics. The School was followed by a very successful Conference, with more than 85 presentations. Plenary lectures were delivered by René de Borst, Dominik Schillinger, Stefanie Elgeti and Emilio Martínez-Pañeda. The closing lecture was delivered by the winner of The Roger Owen Prize for the best Ph.D. thesis. To celebrate the 30th Anniversary of UKACM, a competition was organised, and prizes awarded, for the best pictures related to the Conference posted on social media.

**Fig. 19:**
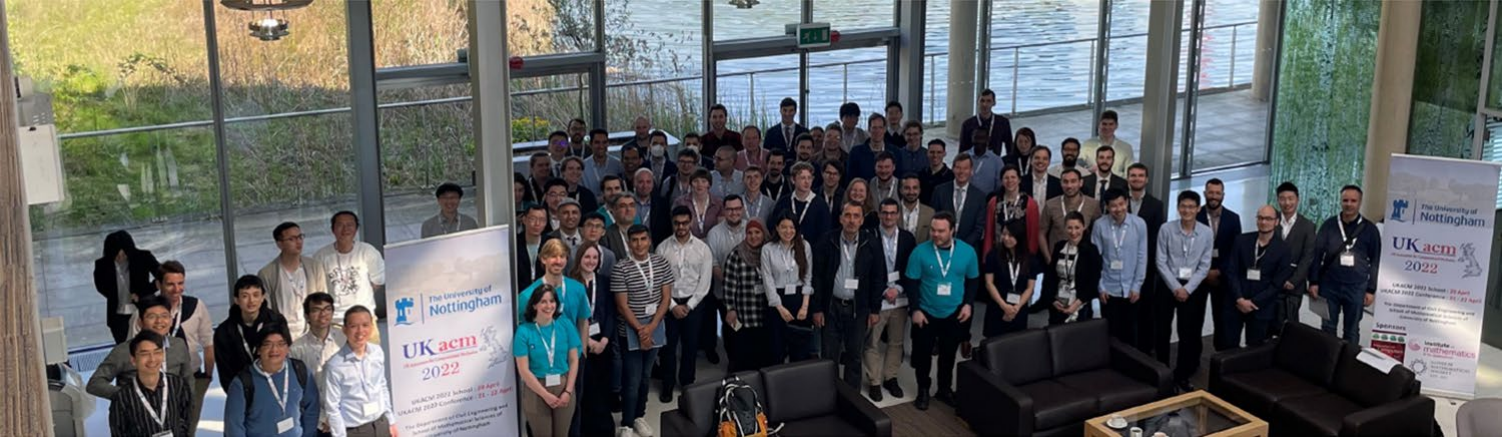
Group photo taken during the 2022 Conference held at Nottingham University. Among those present here are C. H. Lee, E. Martínez-Pañeda, F. Wang, J. Ninic, W. Tan, K. van der Zee, A. Ahangar-Asr, R. Sevilla, R. de Borst, S. Elgeti, D. Schillinger, L. Kaczmarczyk, C. Augarde

The group photograph taken during the 2022 Conference in Nottingham University is shown in Fig. 19.

In 2022, Rubén Sevilla led a negotiation with River Publishers that culminated in an agreement signed between UKACM and the European Journal of Computational Mechanics. This agreement, made the 30th anniversary year of both UKACM and the European Journal of Computational Mechanics, will provide free access to the Journal for UKACM members and sponsorship for all the UKACM Conference awards.

## The future of UKACM

The future of UKACM can be summarised in one word: bright! Not only does the number of Conference attendees continue to increase but, more importantly, the quality of the research presented every year is a testimony to the healthy state of the computational mechanics field in the UK. The main mission of UKACM remains to be a platform for the young generation of UK researchers in the field of computational mechanics. The foundation of a more professional management established in 2015 by Charles Augarde, and maintained by Rubén Sevilla, together with the emphasis on new talent and inclusivity and diversity, offers a unique platform for forthcoming generations. The UKACM Executive Committee and UKACM Board have made major contributions to the continued success of UKACM and they will continue to play a crucial role. It is anticipated that UKACM will continue to grow, enabling talented participants to disseminate their research and early career researchers to be involved in shaping the future of our Association.
